# Supplementary material for: Proteomic and Transcriptomic Analysis for Identification of Endosymbiotic Bacteria Associated with BYDV Transmission Efficiency by Sitobion miscanthi
Source: Plants (Basel). 2022 Dec 2;11(23):3352. doi: 10.3390/plants11233352 (PMC9735544; doi:10.3390/plants11233352)
Supplement: Supplementary file 1 [file plants-11-03352-s001.zip › Non-published material /Table S1. Endosymbiont detected in the Shanxi Taiyuan (STY) geographic population and Henan Dengzhou (HDZ) geographic population of Sitobion miscanthi with and without antibiotic treatment.docx]

**Table S1.** Endosymbiont detected in the Shanxi Taiyuan (STY) geographic population and Henan Dengzhou (HDZ) geographic population of *Sitobion miscanthi* with and without antibiotic treatment.

| Endosymbiont | HDZ-free | HDZ-Amp | HDZ-Rif | STY-free | STY-Amp | STY-Rif |
| --- | --- | --- | --- | --- | --- | --- |
| *Buchnera* | + | + | + | + | + | + |
| PASS1^a^ | - | - | - | - | - | - |
| PASS2 | - | - | - | - | - | - |
| PAUS^b^ | - | - | - | - | - | - |
| PABS^c^ | - | - | - | + | + | + |
| *Rickettsia*1 | + | - | - | + | - | - |
| *Rickettsia*2 | - | - | - | - | - | - |
| *Spiroplasma*1 | - | - | - | + | + | - |
| *Spiroplasma*2 | - | - | - | + | - | - |
| *Wolbachia* | - | - | - | - | - | - |
| *Arsenophonus* | - | - | - | + | + | - |

-free: aphids’ geographic population fed sucrose diet without antibiotic for 48H; -Amp: aphids’ geographic population treated with 50 μg ml^-1^ ampicillin for 48H; -Rif: aphids’ geographic populations treated with 50 μg ml^-1^ rifampicin for 48H; +: strain was examined for endosymbiont; –: not detected.

^a^: γ-*Proteobacteria*, R-type symbiont

^b^: γ-*Proteobacteria,* U-type symbiont

^c^: γ-*Proteobacteria,* T-type symbiont
