# Supplementary material for: Proteomic and Transcriptomic Analysis for Identification of Endosymbiotic Bacteria Associated with BYDV Transmission Efficiency by Sitobion miscanthi
Source: Plants (Basel). 2022 Dec 2;11(23):3352. doi: 10.3390/plants11233352 (PMC9735544; doi:10.3390/plants11233352)
Supplement: Supplementary file 1 [file plants-11-03352-s001.zip › Non-published material /Table S4 Specific primers used in this study.doc]

Table Specific primers used in this study

| Application | Target symbiont | Target gene | Primer name | Primer sequence (5’-3’) | Product size (kb) | References |
| --- | --- | --- | --- | --- | --- | --- |
| PCR | *Buchnera* | 16SrDNA | Buch16S1F | GAGCTTGCTCTCTTTGTCGGCAA | 0.43 | Tsuchida *et al*. 2002 |
|  |  |  | Buch16S1R | CTTCTGCGGGTAACGTCACGAA |  | Tsuchida *et al*. 2002 |
|  |  | 16SrDNA | 16SA1 | AGAGTTTGATCMTGGCTCAG | 0.321 | Fukatsu and Nikoh, 1998 |
|  |  |  | ApisP1 | TTCCAGTGTGGCTGGTTA |  | Tsuchida *et al*. 2002 |
|  | PASS | 16SrDNA | 16SA1 | AGAGTTTGATCMTGGCTCAG | 0.48 | Fukatsu and Nikoh, 1998 |
|  |  |  | PASScmp | GCAATGTCTTATTAACACAT |  | Fukatsu *et al*. 2000 |
|  |  | *groEL* | GroEAF1 | CCTCAAGGCTGTGGCCG | 0.15 | Tsuchida *et al*. 2002 |
|  |  |  | GroEAR1 | TAGGCACCATCTCTGCAAACTC |  | Tsuchida *et al*. 2002 |
|  | PAUS | 16SrDNA | U99F | ATCGGGGAGTAGCTTGCTAC | 0.2 | Sandström et al. 2001 |
|  |  |  | 16SB4 | CTAGAGATCGTCGCCTAGGTA |  | Tsuchida *et al*. 2002 |
|  | PABS | 16SrDNA | PABSF | AGCACAGTTTACTGAGTTCA | 1.66 | Darby et al. 2002 |
|  |  |  | 16SB1 | TACGGYTACCTTGTTACGACTT |  | Fukatsu and Nikoh, 1998 |
|  | *Rickettsia* | 16SrDNA | 16SA1 | AGAGTTTGATCMTGGCTCAG | 0.2 | Fukatsu and Nikoh, 1998 |
|  |  |  | Rick16SR | CATCCATCAGCGATAAATCTTTC |  | Fukatsu *et al*. 2001 |
|  |  | Citrate | CS1 | GGGGGCCTGCTCACGGCGG | 0.36 | Fukatsu and Shimada, 1999 |
|  |  | synthase | CS2 | ATTGCAAAAAGTACAGTGAACA |  | Fukatsu and Shimada, 1999 |
|  | *Spiroplasma* | 16SrDNA | 16SA1 | AGAGTTTGATCMTGGCTCAG | 0.51 | Fukatsu and Nikoh, 1998 |
|  |  |  | TKSsspR | TAGCCGTGGCTTTCTGGTAA |  | Fukatsu and Nikoh, 2000 |
|  |  | *dnaA* | ApDnaAF1 | ATTCTTCAGTAAAAATGCTTGGA | 0.45 | Tsuchida *et al*. 2002 |
|  |  |  | ApDnaAR1 | ACACATTTACTTCATGCTATTGA |  | Tsuchida *et al*. 2002 |
|  | *Wolbachia sp.* | wsp | wspF | GGGTCCAATAAGTGATGAAGAAAC | 0.57 | Kondo *et al*. 2002 |
|  |  |  | wspR | TTAAAACGCTACTCCAGCTTCTGC |  | Kondo *et al*. 2002 |
|  | *Arsenophonus sp.* | 16SrDNA | 16SA1 | AGAGTTTGATCMTGGCTCAG | 0.96 | Fukatsu and Nikoh, 1998 |
|  |  |  | Ars16SR | TTAGCTCCGGAGGCCACAGT |  | Tsuchida *et al*. 2002 |
| qPCR | *Buchnera* | 16SrDNA | Buch-Q-F | GGGAACTCAGAGGAGACTGC | 0.18 | Li *et al*., 2018 |
|  |  |  | Buch-Q-R | TGAGGTTTGCTTGTCTTTGC |  |  |
|  | PABS | 16SrDNA | Hami-Q-F | TGAACAATGTCCCAACTGCT | 0.168 | Li *et al*., 2018 |
|  |  |  | Hami-Q-R | CGCCTCATCTTTCCTGGTAT |  |  |
|  | *Rickettsia* | 16SrDNA | 16SA1 | AGAGTTTGATCMTGGCTCAG | 0.2 | Fukatsu and Nikoh, 1998 |
|  |  |  | Rick16SR | CATCCATCAGCGATAAATCTTTC |  | Fukatsu *et al*. 2001 |
|  | *Arsenophonus sp.* | 16SrDNA | 16SA1 | AGAGTTTGATCMTGGCTCAG | 0.19 |  |
|  |  |  | Ars-Q-R | TAGCTCCGGAGGCCACAGT |  |  |
|  | *Spiroplasma* | 16SrDNA | Spir-Q-F | TGGGATAACTCCGGGGAAACC | 0.18 | Li *et al*., 2018 |
|  |  |  | Spir-Q-R | TGGTAAACCGGTACCCTTCC |  |  |
|  | β-actin | β-actin | β-actin-Q-F | CGTTACCAACTGGGACGATATG | 0.16 | Li *et al*., 2018 |
|  |  |  | β-actin-Q-R | GCGTTCAATGGAGCTTCTGTTA |  |  |
